# Correlation Coefficients for a Study with Repeated Measures

**DOI:** 10.1155/2020/7398324

**Published:** 2020-03-26

**Authors:** Guogen Shan, Hua Zhang, Tao Jiang

**Affiliations:** ^1^Epidemiology and Biostatistics Program, School of Public Health, University of Nevada Las Vegas, Las Vegas, NV 89154, USA; ^2^School of Computer and Information Engineering, Zhejiang Gongshang University, Hangzhou, Zhejiang, China; ^3^School of Statistics and Mathematics, and School of Business, Zhejiang Gongshang University, Hangzhou, China

## Abstract

Repeated measures are increasingly collected in a study to investigate the trajectory of measures over time. One of the first research questions is to determine the correlation between two measures. The following five methods for correlation calculation are compared: (1) Pearson correlation; (2) correlation of subject means; (3) partial correlation for subject effect; (4) partial correlation for visit effect; and (5) a mixed model approach. Pearson correlation coefficient is traditionally used in a cross-sectional study. Pearson correlation is close to the correlations computed from mixed-effects models that consider the correlation structure, but Pearson correlation may not be theoretically appropriate in a repeated-measure study as it ignores the correlation of the outcomes from multiple visits within the same subject. We compare these methods with regard to the average of correlation and the mean squared error. In general, correlation under the mixed-effects model with the compound symmetric structure is recommended as its correlation is close to the nominal level with small mean square error.

## 1. Introduction

Repeated-measure designs are increasingly used in practice to evaluate the trajectory of measures. The Alzheimer's Disease Neuroimaging Initiative (ADNI) study is a longitudinal study to investigate the progression of Alzheimer's disease (AD) [1, 2]. This study evaluates the normal cognitive aging with the focus on mild cognitive impairment (MCI) and early AD. Brain structure and function are two research areas of interest in the ADNI study. As expected, brain structure volumes are often highly associated with results from cognitive tests [3–5]. In a longitudinal study, correlation for repeated measures should be calculated and reported. However, recent articles still only reported the Pearson correlation coefficient that ignores the correlation of outcomes from the same subject. For these reasons, it is important to compare the existing correlations for repeated measures and make recommendations for other researchers to use.

Bland and Altman [6, 7] discussed several approaches to compute correlations for repeated measures. They proposed calculating subject means to compute the Pearson correlation, where subject means eliminate the correlation of outcomes from the same subject. The second approach is to fit a linear regression model with one measure as the dependent variable and the other measure and the subject as the predictor variables. The second approach is similar to the one proposed by Christensen [8] who suggested computing correlation after adjusting for the subject effect [9–12]. In a repeated-measure study, the visit effect is the correlation within the subject. Lipsitz et al. [13] proposed computing partial correlation adjusting the visit effect. When data are correlated, mixed-effects models may be utilized to analyze data while controlling for these additional correlations. Lam et al. [14] were among the first to propose computing correlation between repeated measures under the compound symmetric (CS) correlation structure. Later, Hamlett et al. [15] developed programs to compute correlation under the CS structure by using the commercially available statistical software, SAS. In the work by Lam et al. [14], they also computed the correlation under the autoregressive correlation structure, AR(1). After that, Roy [16] developed SAS macros to compute correlation under the AR(1) structure and compared the correlations for repeated measures under these two correlation structures with limited simulation studies.

The objective of this manuscript is to conduct extensive simulation studies to compare the existing correlations for repeated measures with regard to the average of correlation and the mean squared error (MSE) and identify the correlation method that has the best performance to be used in practice. In addition to the parameter of interest (correlation for repeated measures), there are several nuisance parameters in the variance-covariance matrix: variances, correlations within each outcome, and correlation between outcomes from different visits [17–20]. It is computationally intensive for these comparisons. We have to use supercomputers for simulation studies. However, it is computationally feasible to calculate correlations for an observed data set. We use one example from the ADNI study to illustrate the application of the considered methods to calculate correlation between hippocampal volumes and a neuropsychological assessment to evaluate verbal memory.

We organize this article as follows. In Section 2, we introduce the existing methods to calculate correlations for repeated measures. In Section 3, we conduct extensive Monte Carlo simulation studies to compare the performance of the considered correlations with regard to the average of correlation and the MSE. A real example from the ADNI study is then used to illustrate the application of these correlations. Lastly, we provide conclusions in Section 4 on computing correlation for repeated measures when heterogeneity of correlation is observed across visits.

## 2. Methods

For a repeated-measure study with *n* participants, each participant has several scheduled visits (*m*_*i*_ visits for the *i*-th subject). Suppose *U* and *W* are the two measures in a repeated-measure study and *U*_*ij*_ and *W*_*ij*_ are the outcomes of the *i*-th subject at the *j*-th visit, where *i* = 1, 2,…, *n* and *j* = 1, 2,…, *m*_*i*_. The correlation between *U* and *W*, *ρ*_*UW*_, is the parameter of interest to quantify a relationship between them. Several methods have been proposed to calculate *ρ*_*UW*_, including independence models, partial correlation models, and mixed-effects models.

### 2.1. Independent Assumption

Bland and Altman [6, 7] were among the first to provide methods to compute longitudinal correlation coefficient. One of their approaches assumes the independence between outcomes from the same subject: *U*_*ij*_ ⊥ *U*_*ij*′_ and *W*_*ij*_⊥*W*_*ij*′_. The longitudinal correlation *ρ*_*UW*_ is computed as the Pearson correlation by ignoring the correlation structure from repeated measures. This approach is referred to as the I approach, with the computed correlation as *ρ*_*I*_. This is a naive approach that is easy to apply. Irimata and Li [21] found that *ρ*_*I*_ for a pharmacokinetics data set is very close to other correlations computed from other complicated models.

### 2.2. Subject Means

As suggested by Bland and Altman [6], the correlation can be computed by using the averages at the subject level to eliminate the subject effect in repeated measures. This correlation is able to address the research question whether the average of one measure is related to the average of another. When correlation within each measure is large, *ρ*_*UW*_ at different visits should be similar to each other, and this average correlation model would have good performance. We refer to this correlation approach as the *M* approach with the notation of *ρ*_*M*_.

These two correlations for repeated measures, *ρ*_*I*_ and *ρ*_*M*_, are the Pearson correlation and can be computed by using many statistical software: such as the Proc corr procedure in SAS and the function cor or cor.test in R [22]. The next five correlations are computed from regression models (e.g., mixed-effects models), and we would like to suggest using SAS Proc mixed procedure for implementation. Detailed SAS programs are provided in the Appendix.

### 2.3. Correlation Adjusting for the Subject Effect

Christensen [8] proposed computing correlation for repeated measures by partialling out the subject effect. The subject effect can be removed from the two measures by fitting a multivariate regression model with both measures being the outcomes and the subject ID as the only covariate. The residuals are used to compute the final correlation, which is essentially a partial correlation method for repeated data. This correlation is referred to as the PS correlation that partials out the subject effect, *ρ*_*PS*_.

### 2.4. Correlation Adjusting for the Visit Effect

In the *ρ*_*PS*_ calculation, the correlation between the two measures is included in the multivariate model. In addition to that correlation, another correlation between measures at different visits may be considered. Lipsitz et al. [13] proposed computing partial correlation between outcome and one of the covariates by using this approach. When one of the two measures (e.g., measure *U*) is considered as the dependent variable, the other measure (*W*) is considered as the covariate. The correlation structure between visits is assumed to be compound symmetric. We refer this correlation as the *ρ*_*PVa*_ correlation. We use *ρ*_*PVb*_ for another correlation when *W* is considered as the dependent variable in the model. One of the properties for correlation is *ρ*_*UW*_=*ρ*_*WU*_, but this property is not met here: *ρ*_*PVa*_ is generally not equal to *ρ*_*PVb*_.

### 2.5. Mixed-Effects Model

Let *Y*_*i*_=(*U*_*i*1_, *W*_*i*1_, *U*_*i*2_, *W*_*i*2_,…, *U*_*im*_*i*__, *W*_*im*_*i*__) be the outcomes from the *i*-th subject, with the vector length of 2*m*_*i*_. The complete data can be reorganized in a long format, with the columns subject ID, visit, mtype, and outcome, where mtype = “*U*” for the *U* measure and mtype = “*W*” for the *W* measure. The long format utilizes 2*m*_*i*_ rows for the outcomes from *Y*_*i*_.

The linear mixed-effects model is presented as

(1) $$\begin{array}{c} Y_{i}=X_{i}\beta +Z_{i}b_{i}+\epsilon_{i}, \end{array}$$

where *X*_*i*_ and *Z*_*i*_ are the design matrices for the fixed effect and the random effect, respectively. The random effect *b*_*i*_ follows a multivariate normal distribution *N* (0, *D*), and the measurement error *ϵ*_*i*_ follows a multivariate normal distribution *N* (0, *R*_*i*_). The detailed formula for *D* and *R*_*i*_ may be found in the article by Hamlett et al. [15]. The fixed effect is *β* = (*β*_0_, *β*_*U*_, *β*_*W*_)′, where *β*_0_ is the intercept, and *β*_*U*_ and *β*_*W*_ are the fixed effects of *U* and *W*, respectively. Correlation between *U* and *W* is computed as

(2) $$\begin{array}{c} \rho_{UW}=\text{Corr}\left(U,W\right), \end{array}$$

which is assumed to be independent of the visit.

Each subject has multiple visits, correlation within *U* is Corr(*U*_*ij*_, *U*_*ij*′_)=*ρ*_*U*_^*d*(*j* − *j*′)^, and the correlation within *W* is Corr(*W*_*ij*_, *W*_*ij*′_)=*ρ*_*W*_^*d*(*j* − *j*′)^, where *d* (*j* − *j*′) = 1 for the CS structure and *d* (*j* − *j*′) = |*j* − *j*′| for the AR(1) structure. Since *W*_*ij*_ is correlated with both *U*_*ij*_ and *W*_*ij*′_, therefore, *U*_*ij*_ and *W*_*ij*′_ are correlated and their correlation is assumed to be *δρUW*, where *δ* is a factor which is generally less than 1. Let *σ*_*U*_^2^ and *σ*_*W*_^2^ be the variances of *U* and *W*, respectively. These variances and covariances are used to derive the variance-covariance matrix under the CS structure (see Lam et al. [14] and Hamlett et al. [15]) and that under the AR(1) structure (see Lam et al. [14] and Roy [16]).

## 3. Results

We conduct simulation studies to compare the performance of the considered 7 methods for the correlation between repeated measures for a study with four visits. The mean values of *U* and *W* are assumed to be (2.0, 1.9, 1.7, 1.4) and (0.8, 0.7, 0.6, 0.5), with both measures decreasing as time goes. Such data are commonly available from cognitive tests on elderly population and other studies. The prespecified correlation for repeated measures is *ρ*_*UW*_=0.2, 0.5, and 0.8.

In the simulation studies for the AR(1) structure for the visit effect, the correlation within *U* is Corr(*U*_*ij*_, *U*_*ij*′_)=*ρ*_*U*_^|*j* − *j*′|^, with *ρ*_*U*_=0.2, 0.5, and 0.8, and the correlation within *W* is Corr(*W*_*ij*_, *W*_*ij*′_)=*ρ*_*W*_^|*j* − *j*′|^, with *ρ*_*W*_=0.2, 0.5, and 0.8. The factor *δ* in the correlation between *U*_*ij*_ and *W*_*ij*′_ is assumed to be 0.6 in all simulations. The considered variances are *σ*_*U*_^2^=1 and 3 and *σ*_*W*_^2^=0.5 and 1. The variance-covariance matrix can be separated into two parts: *Z*_*i*_*DZ*_*i*_′ and *R*_*i*_. We assume that a quarter of variance is from *R*_*i*_ and the remaining is from *Z*_*i*_*DZ*_*i*_′. This weight is needed in order to calculate the covariances. For each configuration, we simulate *B* = 2,000 data sets.

**Figure 1:**
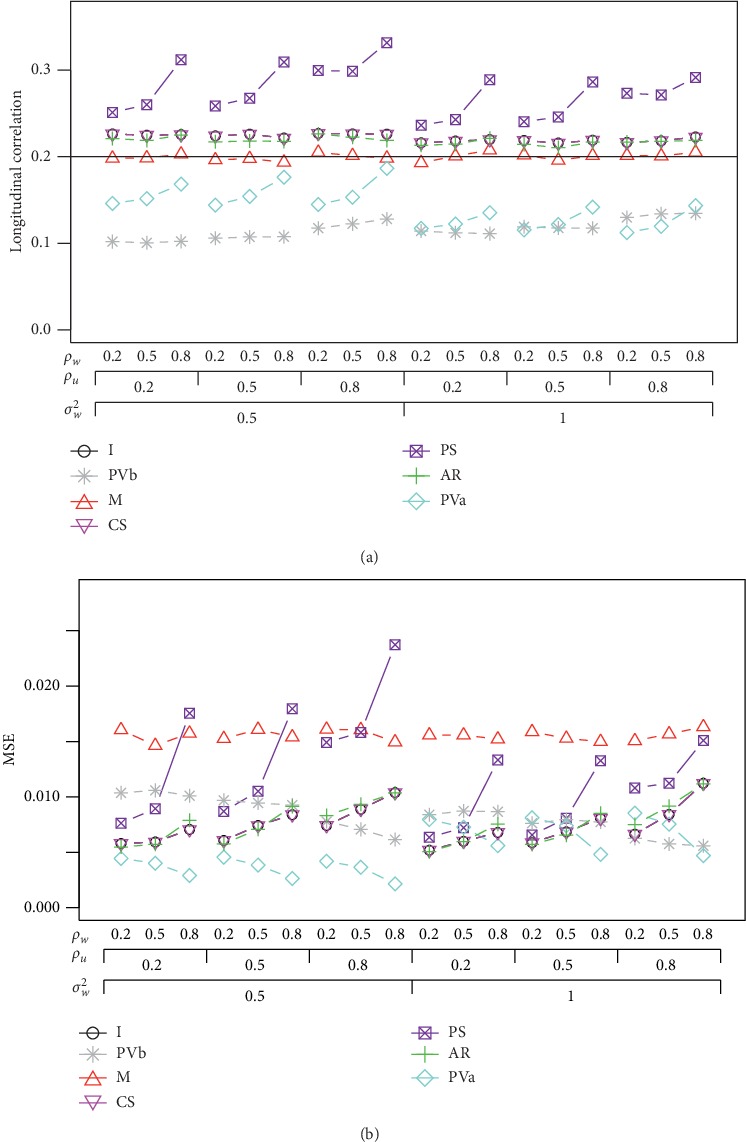
Average correlation and the MSE for the 7 methods under the AR(1) correlation structure when *ρ*_*UW*_ = 0.2, *σ*_*U*_^2^=1, and *n* = 60.

Under the AR(1) structure for the visit effect, Figure 1 presents the average of correlation *ρ*_*UW*_ and the MSE when *ρ*_*UW*_=0.2, *σ*_*U*_^2^=1, and *n* = 60 subjects. The MSE is defined as

(3) $$\begin{array}{c} \text{MSE}=\frac{1}{B}\sum_{b=1}^{B}{\left({\hat{\rho }}_{UW}\left(b\right)-\rho_{UW}\right)}^{2}, \end{array}$$

where ${\hat{\rho }}_{UW}\left(b\right)$ is the estimator of *ρ*_*UW*_ by using the *b*-th simulated data set. It can be seen that the correlations adjusting the visit effect, *ρ*_*PVa*_ and *ρ*_*PVb*_, often underestimate the correlation, while the correlation adjusting the subject effect, *ρ*_*PS*_, always overestimate the correlation. The remaining methods have correlations close to the nominal level. Although *ρ*_*M*_ is the best with the correlation around the nominal level, its MSE is much larger than the ones that have the correlations close to the nominal level. In the calculation of *ρ*_*M*_, each subject only has one outcome for each measure, as compared to multiple outcomes in other correlation calculations. Due to the reduced number of outcomes, the variance of *ρ*_*M*_ is much large that leads to a large MSE. It is noted that *ρ*_*PVa*_ or *ρ*_*PVb*_ could have the lowest MSE in some cases, but their estimated correlations are generally much below the nominal level. For this reason, we exclude *ρ*_*PVa*_ and *ρ*_*PVb*_ in the following simulation studies. When a study has the same number of visits for each subject, the estimated correlation by using the mixed-effects model with the CS structure, *ρ*_*CS*_, is very similar to *ρ*_*I*_ under the independent assumption. The other mixed-effects model correlation *ρ*_*AR*_ has a similar correlation as *ρ*_*CS*_ and *ρ*_*I*_. The MSE of *ρ*_*AR*_ is slightly smaller than the MSEs of *ρ*_*CS*_ and *ρ*_*I*_ when the correlations within *U* or *W* are small, and this trend is reversed when *ρ*_*U*_ and *ρ*_*W*_ are large. Similar results are observed when *σ*_*U*_^2^ is increased to 3.

**Figure 2:**
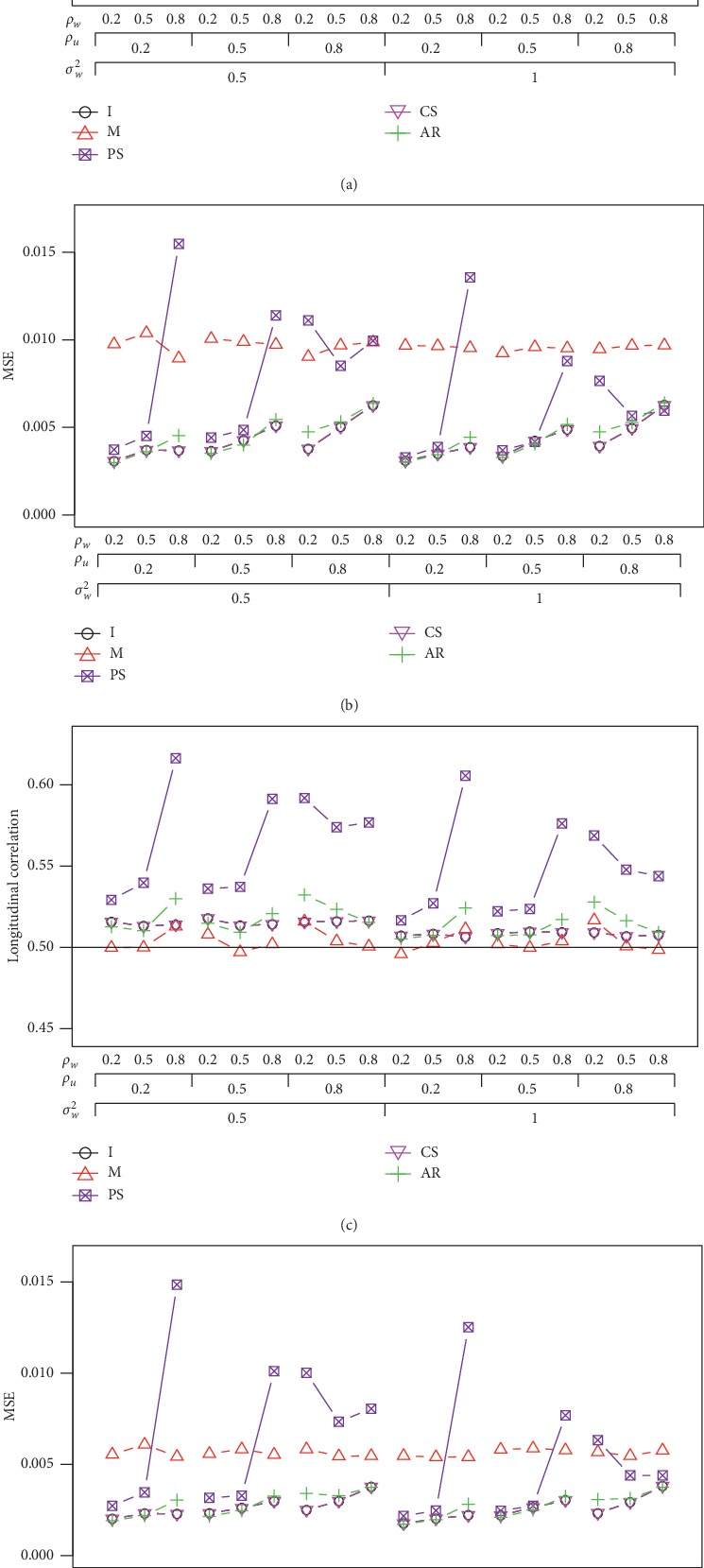
Average correlation and the MSE under the AR(1) correlation structure with *n* = 60 (top) and *n* = 100 (bottom) when *ρ*_*UW*_ = 0.5 and *σ*_*U*_^2^=1.

When *ρ*_*U*_ is increased to 0.5 (the top plot in Figure 2), the averages of *ρ*_*I*_, *ρ*_*CS*_, and *ρ*_*AR*_ are generally above the nominal level, and the first two correlations are closer to the nominal level as compared to the third correlation *ρ*_*AR*_. We also present the correlation estimates when sample size *n* is 100 in Figure 2. It can be seen that the MSEs become smaller as compared to the MSEs in the top plot (Figure 2) when sample size is 60.

**Figure 3:**
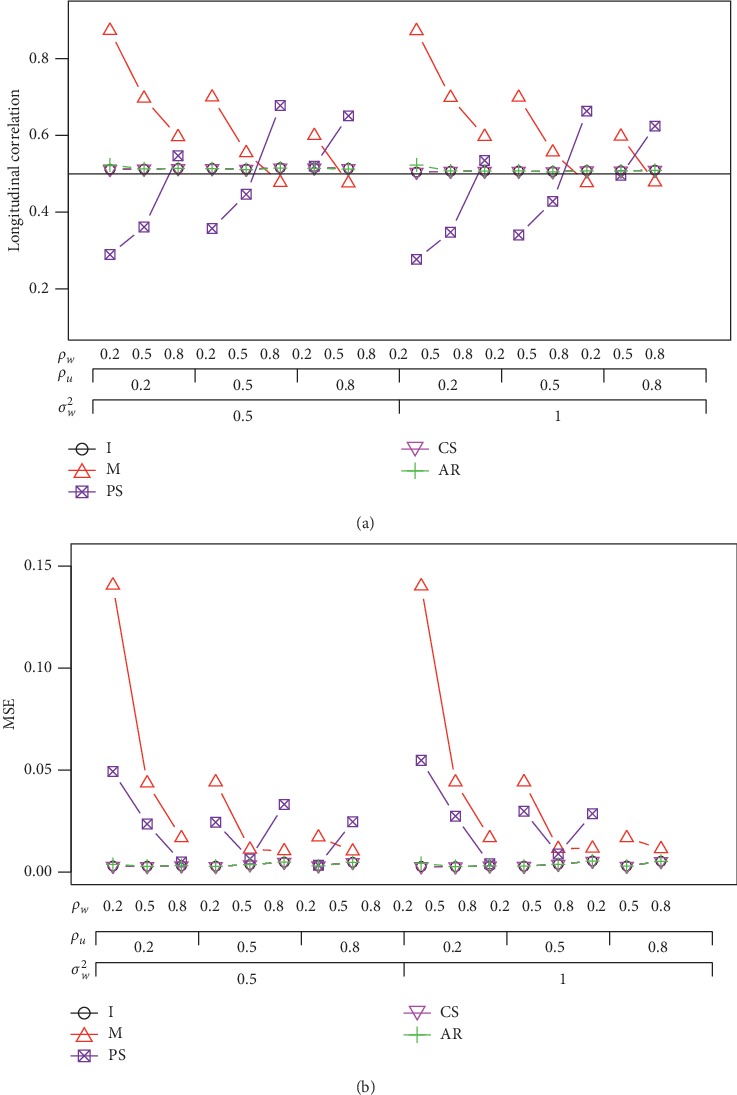
Average correlation and the MSE under the CS correlation structure when *ρ*_*UW*_ = 0.5, *σ*_*U*_^2^=1, and *n* = 60.

Figure 3 shows the results when data sets are simulated under the CS structure given *ρ*_*U*_=0.5, *σ*_*U*_^2^=1, and *n* = 60. Correlation *ρ*_*PS*_ does not perform well with the average correlations much below the nominal level in many configurations. We also found that *ρ*_*M*_ is likely to overestimate the correlation. It seems that *ρ*_*M*_ and *ρ*_*PS*_ have different trajectories as *ρ*_*W*_ increases. Both of these methods do not have satisfactory performance with regard to correlation under the CS structure, although *ρ*_*PS*_ has very good correlation estimates under the AR(1) structure. The other three correlations (*ρ*_*I*_, *ρ*_*CS*_, and *ρ*_*AR*_) have similar good performance with regard to correlation and the MSE. It should be noted that the variance-covariance matrix is not positively defined when *ρ*_*U*_=*ρ*_*W*_=0.8. Therefore, data sets cannot be generated for that configuration. We also simulate data under the unstructured correlation structure and found that *ρ*_*I*_, *ρ*_*CS*_, and *ρ*_*AR*_ are still the best correlation estimates.

**Figure 4:**
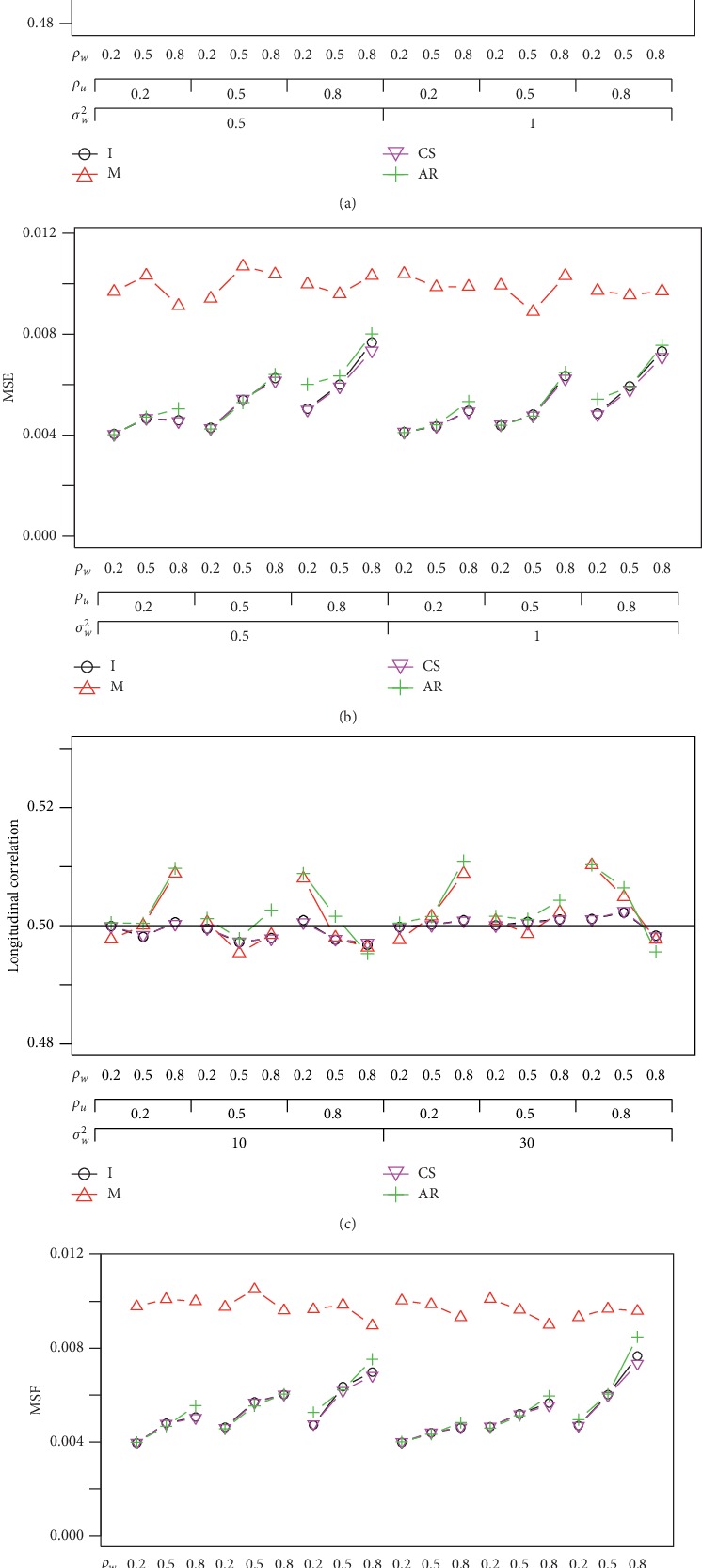
Average correlation and the MSE under the AR(1) correlation structure with a small variance *σ*_*U*_^2^=1 (top) and a large variance *σ*_*U*_^2^=20 (bottom) when *ρ*_*UW*_ = 0.5 and *n* = 60 for a study with unequal numbers of visits (2, 3, or 4 visits).

The aforementioned simulations have data sets that each subject has the same number of visits. In practice, it is possible that the number of visits may not be exactly the same for all subjects. We assume the number of visits is either 2, 3, or 4. Each subject is randomly assigned to have 2, 3, or 4 visits with the same probability. We present the results with *n* = 60 in Figure 4 when variances are small (*σ*_*U*_^2^=1 and *σ*_*W*_^2^=0.5 and 1) and large (*σ*_*U*_^2^=20 and *σ*_*W*_^2^=10 and 30). The MSE of *ρ*_*CS*_ is slightly smaller than that of *ρ*_*I*_, and their biggest difference occurs when both *ρ*_*U*_ and *ρ*_*W*_ are large. *ρ*_*AR*_ is more likely to overestimate the correlation. Although *ρ*_*M*_ has the correlation very close to the nominal level, it has the largest MSE as compared to other correlations. When variance is large, *ρ*_*I*_ and *ρ*_*CS*_ are the best correlations with the estimated correlations much closer to the nominal level as to the configurations with small variances. The mixed-effects model correlation *ρ*_*CS*_ performs slightly better than *ρ*_*I*_ with regard to the average of correlation and the MSE.

### 3.1. Example

We use one data set from the ADNI study to illustrate the application of the considered correlation methods, with 47 participants who had 5-year visits and completed imaging volumes and memory scores. Hippocampal volumes are found to be highly associated with the delayed recall scores from the Rey Auditory Verbal Learning Test (RAVLT delayed recall) [23]. The RAVLT delayed recall has the possible integer score from 0 to 15, which is often used to assess verbal memory. The higher the score is, the better the memory is.

The computed correlations are presented in Table 1. Participants in this data set have the same number of visits. For this reason, *ρ*_*I*_ is very similar to *ρ*_*CS*_. *ρ*_*M*_ is slightly larger than them, and *ρ*_*AR*_ is smaller than them. Correlation adjusted by the subject effect *ρ*_*PS*_ is much smaller than *ρ*_*CS*_. Correlations adjusted by the visit effect highly depend on which variable is considered as the dependent variable in the linear regression model. When hippocampal volumes are used as the dependent variable, the estimated correlation is high (0.686), and it becomes too low (0.016) when RAVLT delayed recalls are considered as the dependent variable.

It was reported by Wang et al. [23] that the Pearson correlation *ρ*_*I*_ between hippocampal volumes and RAVLT delayed recall scores is slightly above 0.4. They also provided the Pearson correlations for each group (AD, MCI, and control) which are all below the correlation using combined samples. The correlation within the control group is the lowest.

From Table 1, RAVLT delayed recall scores always have a larger correlation with left hippocampal volumes than the correlation with right hippocampal volumes for each correlation method. We also add RAVLT immediate recall scores to further illustrate the application of the considered methods. Its correlation with left hippocampal volumes is often larger than its correlation with right hippocampal volumes. The estimated *ρ*_*CS*_ between hippocampal volumes and RAVLT delayed recalls is larger than that between hippocampal volumes and RAVLT immediate recalls.

## 4. Conclusions

From the simulation studies, *ρ*_*I*_ under the independence assumption and *ρ*_*CS*_ using the mixed-effects model with CS variance-covariance structure are shown to have similar correlation estimates when subjects have the same number of visits. But, *ρ*_*CS*_ is appropriate as it models the data properly. The mixed-effects model correlation *ρ*_*CS*_ is recommended for use as its correlation is close to the nominal level with small mean square error.

## 5. Discussions

Lam et al. [14] derived the detailed variance and covariance. The variances *σ*_*U*_^2^ and *σ*_*W*_^2^ and covariance *σ*_*UW*_ are used to calculate *ρ*_*UW*_. These variances and covariance estimates are not exactly the same from the independent model and the mixed-effects model with the CS structure: *σ*_*W*_^2^=16.6846 in the *ρ*_*I*_ calculation and 16.6136 from the CS model. Because these estimated variances and covariance are very close between these two methods, the final estimated correlations are very similar. When a study has different number of follow-up for each participant, *ρ*_*I*_ and *ρ*_*CS*_ differ from each other [18, 24–26]. For a study with some possible outliers as seen in the data testing association between pH and PaCO_2_ [6], their difference is substantial. We provide the SAS programs by using that example in the Appendix.

When CS or AR(1) correlation structure for the visit effect is applied in the mixed-effects models [10, 25, 27], the computed correlation is the same at different visits. In the observation of the heterogeneity of correlations at different visits, the unstructured correlation may be considered for the visit effect. Under the heterogeneity assumption, correlation can be computed at each visit from a mixed-effects model [28–30]. This brings some challenges to explain the results, such as the overall correlation and the trend of correlation. Alternatively, when a study has a monotonic relationship between correlation and visit, one may include an additional predictor: visit, in the statistical model, to calculate a monotonic correlation for repeated measures.

## Figures and Tables

**Table 1:** Correlation between hippocampal volumes and RAVLT delayed recall scores using 47 participants with 5 visits from the ADNI study.

	*ρ*_*I*_	*ρ*_*M*_	*ρ*_*PS*_	*ρ*_*PVa*_	*ρ*_*PVb*_	*ρ*_*CS*_	*ρ*_*AR*_
Left hippocampal and RAVLT delayed recall scores	0.421	0.468	0.151	0.016	0.686	0.421	0.392
Left hippocampal and RAVLT immediate recall scores	0.352	0.421	0.208	0.023	0.447	0.365	0.399
Right hippocampal and RAVLT delayed recall scores	0.361	0.398	0.149	0.014	0.652	0.361	0.327
Right hippocampal and RAVLT immediate recall scores	0.316	0.373	0.211	0.021	0.443	0.335	0.343

## Data Availability

The data used in preparation of this article were obtained from the Alzheimer's Disease Neuroimaging Initiative (ADNI) database (http://adni.loni.usc.edu). As such, the investigators within the ADNI contributed to the design and implementation of ADNI and/or provided data but did not participate in analysis or writing of this report. A complete listing of ADNI investigators can be found at http://adni.loni.usc.edu/wp-content/uploads/how_to_apply/ADNI_Acknowledgement_List.pd.

## Supplementary Materials

Supplementary Materials Supplement is the R code. SAS programs: correlation for repeated measures from 8 critically ill patients: pH and PaCO_2_ [6].
