# Supplementary material for: Correlation Coefficients for a Study with Repeated Measures
Source: Comput Math Methods Med. 2020 Mar 26;2020:7398324. doi: 10.1155/2020/7398324 (PMC7136761; doi:10.1155/2020/7398324)
Supplement: Supplementary Materials — Supplement is the R code. SAS programs: correlation for repeated measures from 8 critically ill patients: pH and PaCO2 [6]. [file 7398324.f1.pdf]

Supplementary

SAS programs: correlation for repeated measures from the 8 critically ill patients: pH and PaCO2 [3].

```
data data_wide;
input ID visit U W @@;
cards;
1 1 6.68 3.97  1 2 6.53 4.12  1 3 6.43 4.09  1 4 6.33 3.97  2 1 6.85 5.27  2 2 7.06 5.37  2 3 7.13 5.41  2 4 7.17 5.44
3 1 7.40 5.67  3 2 7.42 3.64  3 3 7.41 4.32  3 4 7.37 4.73  3 5 7.34 4.96  3 6 7.35 5.04  3 7 7.28 5.22  3 8 7.30 4.82
3 9 7.34 5.07  4 1 7.36 5.67  4 2 7.33 5.10  4 3 7.29 5.53  4 4 7.30 4.75  4 5 7.35 5.51
5 1 7.35 4.28  5 2 7.30 4.44  5 3 7.30 4.32  5 4 7.37 3.23  5 5 7.27 4.46  5 6 7.28 4.72  5 7 7.32 4.75  5 8 7.32 4.99
6 1 7.38 4.78  6 2 7.30 4.73  6 3 7.29 5.12  6 4 7.33 4.93  6 5 7.31 5.03  6 6 7.33 4.93
7 1 6.86 6.85  7 2 6.94 6.44  7 3 6.92 6.52
8 1 7.19 5.28  8 2 7.29 4.56  8 3 7.21 4.34  8 4 7.25 4.32  8 5 7.20 4.41  8 6 7.19 3.69  8 7 6.77 6.09  8 8 6.82 5.58
;
run;

data data_long (drop=U W i);
set data_wide;
array var[2] U W;
do i = 1 to 2;
mtype = i;
Outcome = var[i];
output;
end;
run;

proc format;
value mtypea
1="U"
2="W"
;
run;
data data_long;
set data_long;
format mtype mtypea.;
run;

/*CS correlation, mixed-effects model*/

proc mixed data=data_long method=ml;
class ID mtype visit;
model Outcome = mtype / solution ddfm=kr;
random mtype / type=un subject=ID v vcorr;
repeated mtype visit / type=un@cs subject=ID;
ods output VCorr=VCorr ConvergenceStatus=CSraw CovParms=CovParms FitStatistics=AICraw;
run;

/*AR correlation, mixed-effects model*/
```

```
proc mixed data=data_long method=ml;
class ID mtype visit;
model Outcome = mtype / solution ddfm=kr;
random mtype / type=un subject=ID v vcorr;
repeated mtype visit / type=un@ar(1) subject=ID;
ods output VCorr=VCorr ConvergenceStatus=CSraw CovParms=CovParms FitStatistics=AICraw;
run;

/*Approach 1, I correlation*/

proc corr data=data_wide;
var U W ;
run;

/*Approach 2, naive approach*/

proc summary data=data_wide;
var  U W ;
by ID;
output out=corrB mean(U)=avg_x mean(W)=avg_y;
run;

proc corr data=corrB;
var avg_x avg_y;
run;

/*Approach 3, Partial correlation adjusting for patient effect*/

proc mixed data=data_long method=ML;
 class mtype ID visit;
 model Outcome= mtype*ID / noint notest s;
 repeated mtype / subject=id(visit) type=un;
ods output VCorr=VCorr ConvergenceStatus=CSraw CovParms=CovParms FitStatistics=AICraw;
run;

/*Approach 4, Partial correlation by Lipsitz, y=x*/

proc mixed data=data_wide method=ml;
class ID visit;
model W =U / solution ddfm=kr;
repeated visit / type=CS subject=ID;
ods output SolutionF=out4a Dimensions=out4na;
run;

proc mixed data=data_wide method=ml;
class ID visit;
model U =W / solution ddfm=kr;
repeated visit / type=CS subject=ID;
ods output SolutionF=out4b Dimensions=out4nb;
run;
```
